# Cross-talk between cancer stem cells and immune cells: potential therapeutic targets in the tumor immune microenvironment

**DOI:** 10.1186/s12943-023-01748-4

**Published:** 2023-02-21

**Authors:** Bo Wu, Xiang Shi, Meixi Jiang, Hongxu Liu

**Affiliations:** 1 Department of General Surgery, Cancer Hospital of China Medical University, Liaoning Cancer Hospital & Institute, Shenyang, 110042 China; 2 Department of Thoracic Surgery, Cancer Hospital of China Medical University, Liaoning Cancer Hospital & Institute, Shenyang, 110042 China; 3 Department of Neurology, The Fourth Affiliated Hospital, China Medical University, Shenyang, 110032 China

**Keywords:** Cancer stem cells (CSCs), Immune cells, Tumor immune microenvironment (TIME)

## Abstract

Ongoing research has revealed that the existence of cancer stem cells (CSCs) is one of the biggest obstacles in the current cancer therapy. CSCs make an influential function in tumor progression, recurrence and chemoresistance due to their typical stemness characteristics. CSCs are preferentially distributed in niches, and those niche sites exhibit characteristics typical of the tumor microenvironment (TME). The complex interactions between CSCs and TME illustrate these synergistic effects. The phenotypic heterogeneity within CSCs and the spatial interactions with the surrounding tumor microenvironment led to increased therapeutic challenges. CSCs interact with immune cells to protect themselves against immune clearance by exploiting the immunosuppressive function of multiple immune checkpoint molecules. CSCs also can protect themselves against immune surveillance by excreting extracellular vesicles (EVs), growth factors, metabolites and cytokines into the TME, thereby modulating the composition of the TME. Therefore, these interactions are also being considered for the therapeutic development of anti-tumor agents. We discuss here the immune molecular mechanisms of CSCs and comprehensively review the interplay between CSCs and the immune system. Thus, studies on this topic seem to provide novel ideas for reinvigorating therapeutic approaches to cancer.

## Introduction

Many therapeutic modalities have been developed, which are currently used to treat cancer, such as surgery, radiation, chemotherapy and targeted therapies, but the risk of recurrence remains high [1, 2]. Studies have shown that the proliferation and spread of tumor cells are related to the presence of stem-like cells within the tumor, which are collectively referred to cancer stem cells (CSCs) [3]. The existence of this cell type was first reported in acute myeloid leukemia (AML) [1]; subsequently, the presence of CSCs is also reported in different types of solid tumors, containing brain, breast, lung, liver, pancreas, colon and prostate cancer [4, 5]. These cells are capable of differentiation, self-renewal, tumorigenesis and chemoresistance [6]. CSCs are also capable of controlling the role of immune cells, containing T cells, B cells, NK cells as well as macrophages [7]. The tumor microenvironment is resulted from the presence of immune checkpoint inhibitors, such as programmed death-1/programmed cell death ligand (PD-L1), cluster of differentiation 47 (CD47), T cell immunoglobulin and mucin-containing domain-3 (TIM3), lymphocyte activation gene 3 (LAG3) and cytotoxic T-lymphocyte antigen-4 (CTLA4) [8–10].

The contact between CSCs and immune cells is mediated not only through immune targets, but also through EVs that enable the transfer of large biomolecular cargos among different types of cells. CSCs regulate the composition of TME through the release of EVs and various soluble factors, including cytokines, chemokines, growth factors, metabolites and hormones [11–13]. Several factors are involved in the cross-talk between CSCs and the tumor microenvironment, such as interleukins (ILs) (IL-6, IL-8 and IL-1β), matrix metalloproteinases (MMPs), vascular endothelial growth factor (VEGF) as well as transforming growth factor beta 1 (TGF-β1) which can be freely released into the extracellular space or encapsulated in EVs [14–20]. Given the important immunomodulatory role of CSCs, further scientific studies are still required to evaluate the extent of the clinical impact of CSCs.

The current review primarily concentrates on the recent advances in the crosstalk between CSCs and immune cells, immune checkpoint molecules and EVs in the TME, together with the possible mechanisms of CSCs induced immune suppression in accordance with the above-mentioned interactions. In addition, we show the present understanding of the origins, activators, heterogeneity and plasticity of CSCs. In the end, we present major CSCs-based targeted immunotherapeutic strategies that can probably improve anti-tumor immunity in the TME and show several potential research directions in the future.

## Development of cancer stem cells

### Intrinsic features: genetic and epigenetic

Cancer stem cells (CSCs) stand for a small subpopulation of the tumor and possess self-renewal properties [2, 21–23]. CSCs undergo asymmetric division, giving rise to two different cell types with distinct cellular fates: one retains stem cell-like features keeping the capacity for self-renewal, whereras the other transforms into specialized progenitor cells with the capacity to generate proliferating tumor cells and populate the tumor mass [24]. Several surface markers of CSCs have been identified, including EpCAM, CD44 and CD133, which provide a possible identification method of CSCs in the tumor stroma (Table 1) [25–34].

**Table 1:** Characterization of surface markers of CSC in the tumor immune microenvironment

CSCs	Phenotypes of CSCs	Immune cell Involving CSCs	Immune mechanism of CSCs	Ref
EpCMA	Liver CSCs	NK cell;	The high expression of EpCAM + cells resulting in resistance to NK cell-mediated cytotoxicity	[26]
CD44	SCCHN CSCs	Treg cells; MDSC;	CD44 + cells inhibit T-cell proliferation, Treg cells and MDSC	[27]
CD44	Lung CSCs	B cell; CD4 + T cell; Neutrophil; DC;	CD44 was associated with PD-L1 and infiltration of immune cells, and was a negative prognostic factor for predicting worsed OS in lung adenocarcinoma	[28]
CD44 + /CD133 +	Pancreatic CSCs	CD8 + T Cell;	CD44 + /CD133 + CSCs are associated with low CD8 + T cell infiltration and high PD-L1 expression Level	[29]
CD44 + CD90 +	SCLC CSCs	CD8 + T Cell;	The interaction between CD44 + CD90 + CSC-like cells and T cells led to the upregulation of checkpoint molecules PD-1, CTLA-4, TIM-3, and LAG3	[30]
CD90	Pancreatic CSCs	Monocyte; Macrophage;	The CD90 highly expressed population in PDAC cells harbors high stemness features and tumorigenicity. Notably, CD90 acts as an anchor for monocyte/macrophage adhesion, providing immunosuppressive features	[31]
CXCR4	OSCC CSCs	CAF; TAM; Monocytes;	CAF effectively attracts monocytes via the CXCL12/CXCR4 pathway and induces their differentiation to M2 macrophages	[32]
CD166	Lung CSCs	DC vaccine	Dendritic cell vaccination significantly decreased percentage of CD166 + CSC.This anticancer stemness effect was attributed to the immune-stimulatory effect as indicated by increased percentage of CD83 + and CD8 + cells, upregulation of Il-12, and downregulation of TGF-β, CTLA-4, PD-L1 and FOXP3 gene expression compared to lung cancer control group	[33]
SOX2	Colorectal CSCs	CD8 + T cell; Treg cells;	The prognostic value of the SOX2 cancer stem-like cell marker in colon cancer is modified by expression of immune-cell related factors FoxP3 and PD-L1	[34]
Nanog	Colorectal CSCs	CD8 + T cell;	Inhibition of Nanog in a murine model of colon cancer rendered tumor cells susceptible to immune-mediated clearance and led to successful, long-term control of the disease	[35]

*SCCHN* Squamous cell carcinoma of the head and neck, *SCLC* Small cell lung cancer, *OSCC* Oral squamous cell carcinoma, *NK* Natural killer, *Treg cells* Regulatory T cells, *MDSC* Myeloid-derived suppressor cells (MDSC), *DC* Dendritic cell, *CAF* Cancer-associated fibroblast, *TAM* Tumor-associated macrophage

Intrinsic heterogeneity includes genetic and epigenetic alterations that promote oncogenic activity [1, 35]. Genetic and epigenetic alterations make an integral function in promoting tumor development, progression, survival and therapeutic resistance to treatment. The plasticity of cancer stem cells allows phenotypic switching between CSCs and non-CSCs states in response to environmental signals and is ruled by intrinsic factors [36–39]. Maintenance of cancer stem cell plasticity makes a necessary role in stimulating the growth and survival of tumor cells. The maintenance of the cancer stem cell state can be controlled by genomic changes (chromosomal amplifications, deletions, rearrangements and DNA mutations), epigenetic modifications and microenvironmental cues [40]. In contrast to genetic changes, epigenetic reprogramming facilitates adaptation and resistance to treatments, thus, greatly influencing cellular fate decisions. Similarly, genetic and epigenetic modifications involved in the signaling pathways can promote the stemness of CSCs.

### Cell signaling pathways regulating cancer stem cells

Numerous signaling pathways are activated in CSCs, including Wnt/β-catenin, Notch, Hedgehog (Hh), Nuclear factor kappa B (NF-κB), Yes-associated protein (YAP) and Integrins, making vital functions in controlling cell survival, growth, differentiation and self-renewal. Several components of the cell signaling pathways were found to be genetically altered in CSCs. All these genetic alterations lead to epigenetic reprogramming causing deregulation of many signaling pathways, which collectively determine the fate of CSCs present within the tumors.

The canonical Wnt/β-catenin signaling pathway is considered to be a vital regulator of tumor cell plasticity [41]. Activation of the canonical Wnt signaling pathway can be regulated by the transcription factor β-catenin [42, 43]. Wnt/β-catenin is a signaling pathway which regulates cell proliferation, differentiation, apoptosis and tissue homeostasis [44–46], whereas aberrant Wnt/β-catenin signaling enhances the expression of surface markers of CSCs and promotes self-renewal, localization within specialized niches and other related CSCs properties [47].

The Notch signaling pathway regulates stem cell differentiation and self-renewal [48–50]. Aberrant Notch signaling stimulates self-renewal of CSCs in ovarian, breast, and hepatocellular carcinoma (HCC) [38]. Epigenetic analysis of osteosarcoma cells indicates that leukemia inhibitory factor (LIF) is associated with the activation of NOTCH1 signaling through lysine 27 of histone H3 (H3K27 me3) demethylation, inducing the expression of "stemness" related genes, sphere formation, self-renewal as well as metastasis [51–53].

In CSCs, the hedgehog (Hh) signaling pathway has been engaged in driving tumor growth, invasion and tumor recurrence following therapeutic intervention [54, 55]. In colorectal cancer, cancer-initiating cells express the indian hedgehog (IHH) gene, which is present in a bivalent state and contributes to the maintenance of colorectal cancer-initiating cells [54, 56]. In gastric adenocarcinoma, increased promoter methylation of transcription factors CDX1/2 and KLF5, which are the downstream targets of the sonic hedgehog (SHH) signaling, caused reduced expression of CDX1 and KLF5 and elevated expression of CDX2. Elevated expression of CDX2 was related to lymph node metastases in patients [55]. Likewise, DNA hypermethylation of the CpG bank of the SHH gene leads to the loss of the expression of the SHH gene in invasive uroepithelial carcinoma [57].

Yes-associated protein (YAP) and transcriptional coactivator with PDZ-binding (TAZ) are transcriptional co-activators, which are upregulated in many cancer types [58–61]. A splice variant of the α6 cytoplasmic structural domain (α6β1) of integrin was discovered to be capable of activating TAZ, causing the transcription of genes related to self-renewal [59]. In prostate cancer, over-expression of α3 integrins in drug-resistant cancer cells led to the inhibition of metastasis, which occurred via the inhibition of Rho GTPase activity by Abl kinase in the Hippo oncogenic signaling pathway [62]. However, in glioblastoma, α3 expression was discovered to be associated with tumor invasion and metastasis via the activation of the extracellular signal-regulated kinase 1/2 (ERK1/2) signaling pathway [63].

Integrins refer to heterodimeric cell surface receptors that promote cell proliferation, differentiation, adhesion to extracellular matrix (ECM) and migration by sensing the cellular microenvironment [64–67]. Overexpression of integrins in different cancer types has been documented, and a variety of peptide ligands against integrins have been developed for targeted therapies [68, 69]. The alphavbeta3 (αVβ3) integrin has been implicated in developing resistance to receptor tyrosine kinase inhibitors [60]. Increased expression of αVβ3 integrin also was discovered in lung tumors that mediated resistance to erlotinib [70]. While in another research, integrin α6 was strongly denoted in glioblastoma cells [71]. Currently, α6 is used as a biomarker for the identification of cancer stem cells [72]. In addition, integrins can also regulate cellular signaling events facilitating extracellular signaling events.

Taken together, signaling pathways are deterministic in the establishment of stemness traits. Moreover, the survival of cancer stem cells can also be dependent on tumor microenvironment events in the local niche of the tumor that help them to remain in a quiescent state or switch to a proliferative state. Significantly, there exists a growing body of evidence showing that a favourable environment plays a vital role in dedifferentiating tumor cells into CSCs. Besides, further identification of more detailed microenvironmental signals supporting or determining the stemness is of paramount importance to propose better intervention strategies.

## Immunomodulatory traits of CSCs and tumor microenvironment

In many cancer types, tumors consist of rare subpopulations of CSCs that differ in cellular phenotype, gene expression pattern and functional characteristics [73]. The tumor microenvironment can regulate the development of cancer stem cells [1, 74, 75], which includes the ECM and non-tumor cells present in the tumor stroma, like cancer-associated fibroblasts (CAF) and endothelial cells, exerts a vital function in the progression of the tumor. Stromal cells can regulate the activity of CSCs via paracrine signaling. For example, hepatocyte growth factor (HGF) secreted by myofibroblasts activates Wnt signaling pathway and induces dedifferentiation of non-CSCs into CSCs [76]. Likewise, the vasculature of the tumor microenvironment supports carcinogenesis and provides a specialized ecological niche for CSCs. It was shown that endothelial cells induce CSC phenotype in colon cancer by producing Notch ligand Delta-like ligand 4 (DLL4) [77]. Endothelial cells secrete growth factors that induce stem cell phenotype in glioblastoma [78, 79]. In addition, CSCs can even create their own ecological niche by trans-differentiation into endothelial progenitor cells [80, 81], providing CSCs with the necessary growth factors [80, 82]. Another perspective of the microenvironment possessing the power to influence CSCs behavior is the immune cells. Therefore, a better understanding of the interaction between CSCs and immune cells may provide potential new approaches to develop therapeutic interventions for tumors.

## Immune cells-targeted immunotherapy for CSCs

### Interaction between CSCs and immune cells in the TME

The CSCs niche maintains the state and plasticity of cancer cells and protects them from immune cell attack [26, 83–92]. The persistent interaction of cancer stem cells with the tumor microenvironment confers the ability to avoid recognition and eradication by immune cells, ensuring their survival and development [93, 94]. Therefore, understanding the capacity of cancer cells to circumvent immune evasion is a prerequisite to better understanding the immunobiology of CSC and thus developing more effective therapeutic approaches.

Dendritic cells (DCs) refer to the primary antigen-presenting cells (APCs), presenting tumor-associated antigens (TAAs) on major histocompatibility complex (MHC)-I molecules, thereby activating immune responses. CSCs can either impair the production of mature DCs or enhance the number of tolerogenic DCs by secreting TGF-β1 [95], leading to the downregulation of MHC-II expression as well as the production of CD80, CD86 costimulatory molecules [96–98]. CD105 expressing CSCs secrete EVs carrying MHC-I and human leukocyte antigen G (HLA-G), which impair the maturation of DCs through the signal transducer and activator of transcription 3 (STAT3) signaling pathway [99, 100]. The interaction between C-X-C motif chemokine ligand (CXCL)-12 on regulatory dendritic cells (DCregs) and C-X-C motif chemokine receptor (CXCR)-4 receptor on CSCs contributes to the maintenance of the self-renewal property of CSCs [101]. Furthermore, CXCL1^+^ DC-regs induce stemness signaling in CD133^+^ colon cancer cells to facilitate metastatic capacity [102].

The interaction of tumor-associated macrophages (TAMs) with CSCs confers the emergence of an immunosuppressive TME [103]. Ecotopes of CSCs are enriched in ILs, ECM, TGF-β and periostin that facilitate macrophage recruitment and macrophage polarization [85, 104]. The expression of periostin on the cell membrane of CSCs recruits monocytes from the vasculature [85] and converts monocytes into TAMs in the TME to support the activity and survival of CSCs. TGF-β1 promotes the generation of EpCAM^+^ CSCs, which facilitate HCC invasion and metastasis by triggering epithelial-mesenchymal transition (EMT) [105]. Furthermore, TAMs trigger the over-expression of CD47 on pancreatic [106], HCC [107] and leukemic [108] stem cells. CD47 on CSCs binds to SIRPα on macrophages protecting CSCs from immune cell-mediated phagocytosis. In addition to this, secreted factors from TAMs stimulate the expression of immune checkpoints, including PD-L1 [109]. Overall, the cross-talk between CSC and TAM induces the immunosuppressive TME, which supporting the survival of CSCs and complicates tumor eradication after immunotherapy.

Myeloid-derived suppressor cells (MDSCs) secrete cytokines and chemokines to reduce the efficacy of immunotherapy [110]. The mammalian target of rapamycin (mTOR) signaling in CSCs promotes the infiltration and aggregation of MDSCs at tumor sites [110]. In melanoma, CD133^+^ CSCs activate TGF-β1 expression and recruit immunosuppressive MDSCs in the tumor site [111]. In addition, TIM-3/Galectin 9 (Gal-9) expressed on the surface of leukemic stem cells (LSCs) elevates the number of infiltrating MDSCs and TAMs, leading to impaired anti-tumor immune responses [112]. Similarly, MDSCs induce stemness in CSCs through upregulation of piRNA-823 [113]. Moreover, MDSCs secrete exosome S100A9 that enhances the activity of signal transducer and activator of STAT3/noncanonical nuclear factor-kappaB (NF-κB) signaling [114] and the production of prostaglandin E2 (PEG-E2) [115], promoting cancer cell stemness and survival. These findings suggest that CSCs-MDSCs interactions reshape the stemness of CSCs, leading to tumor growth and progression.

The cross-talk of Tregs with CSCs promotes the formation of immunosuppressive TME. PD-L1 and TGF-β1 expressed by CSCs mediate Tregs infiltration in glioblastoma [116]. Similarly, CSCs secrete CCL1 to recruit Tregs, producing TGF-β1 and IL-17 to stimulate the self-renewal capacity of CSCs [117–119]. Gastric CSCs facilitate the development of cancer stem cells through STAT3 signaling pathway while protecting CSCs from being recognized by T cells [120]. Tregs derive VEGF to maintain the survival, stemness and self-renewal of CSCs under hypoxia conditions [82]. Furthermore, Tregs secreted cyclooxygenase 2 (COX-2) hinders the function of effector T cells in a PEG-E2-dependent mechanism, verifying that the interactions between CSCs and Tregs promote immune escape, leading to the failure of cancer immunotherapy [121].

In general, T cells recognize TAAs on the surface of APCs as MHC-peptide complexes. However, CSCs can downregulate the expression of MHC-I [122] and TAAs [91], induce the expression of allelic variants of MHC-1 [123, 124] and upregulate the expression of immune checkpoints, including PD-L1 [125], to evade immune surveillance and recognition by anti-tumor immunity. Besides, downregulation of MHC-1 expression influences CD8^+^ T cell activation [126]. EMT/β-catenin signaling in CSCs regulates the glycosylation and stabilization of the immune checkpoint PD-L1, thus, evading T cell immune surveillance [127]. In a hypoxia environment, CSCs induce the expression of VEGF, PD-L1 and TIM-3 [128]. During the development and metastasis of human neural crest cells (HNCCs), CD276^+^ CSCs are found to be located at infiltrating tumor sites and evade anti-tumor immunity by hindering the infiltration of CD8^+^ T cells [129, 130]. Prostate CSCs inhibit T cell proliferation and cytokine production via Gal-3 expression, thus, protecting CSCs from cytotoxic T cells mediated lysis [131]. Furthermore, quiescent CSCs protect the ability of T cells to recognize and lysis of tumor cells by downregulating NLR family CARD domain containing 5 (NLRC5) trans-activator which belongs to the MHC class I mediated immune responses [132].

**Fig. 1:**
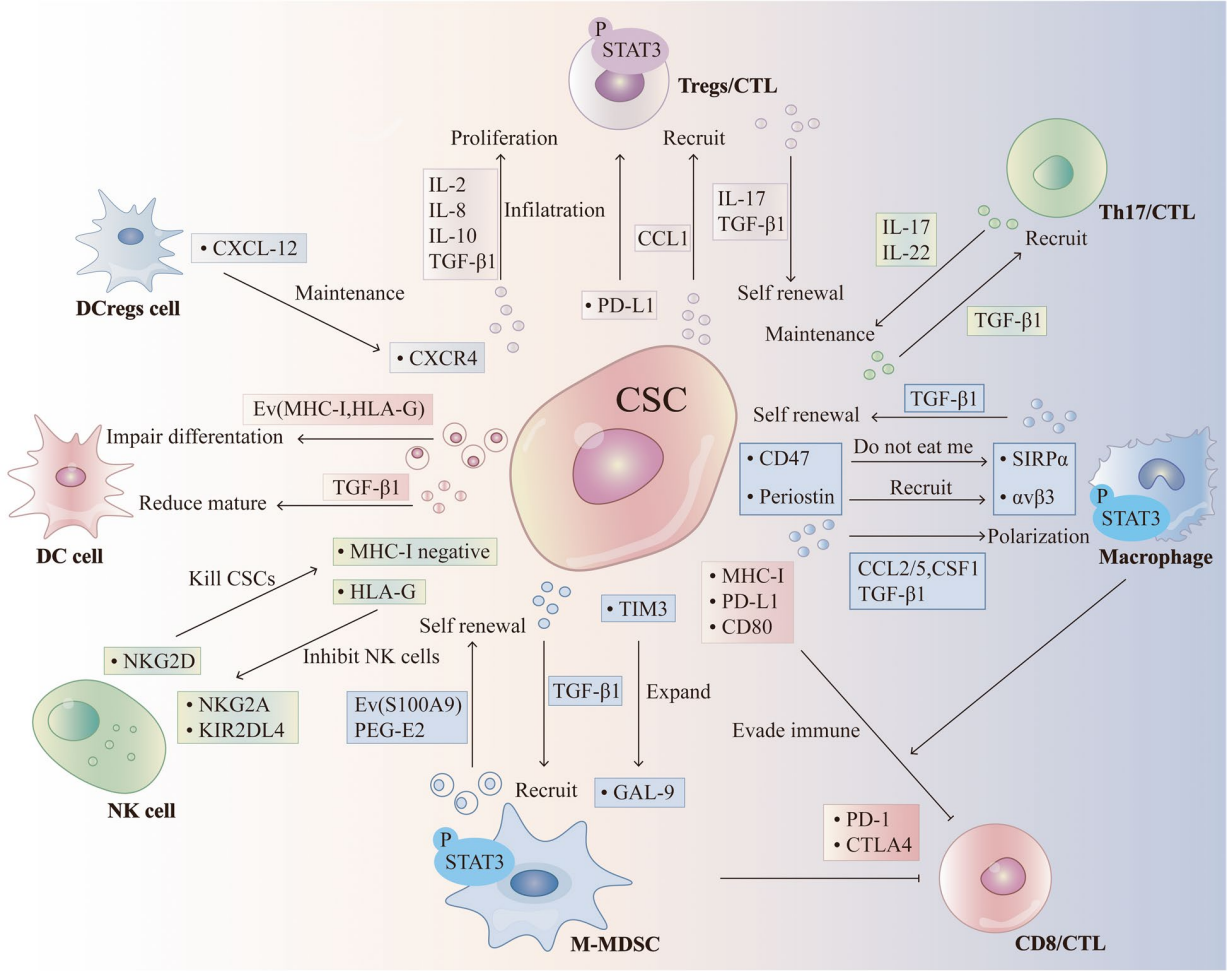
CSCs interfere with immune cell activity directly or through cytokines. CSCs suppress or evade antitumorigenic T cells in part by immune checkpoint (MHC-I, PD-L1 and CD80). CSCs reduce DCs mature and differentation via TGFβ and Ev (MHC-I, HLA-G). NKG2DL are able to kill MHC-I negative CSCs in an APC-independent manner. NK cells inhibitory ligands KIR2DL4 and NKG2A interact with HLA-G on CSCs and directly inhibits NK cells activation. CSCs further drive recruitment and polarization of TH17 cells and Treg cells by the combination of CCL-1, IL-2, IL-8, IL-10 and TGF-β1. Tregs produce TGF-β1 and IL-17 to promote self-renewal capacity, stem cell markers, and EMT toward tumor progression and invasion. CSCs also derived PD-L1 mediate the infiltration of Tregs. An additional layer of regulation of T cell activity is mediated indirectly by immunosuppressive myeloid cells, including macrophages and monocytic myeloid-derived suppressor cells (M-MDSCs). This effect partially depends on CSF1, CCL2, CCL5, TGF-β1 and PEG-E2 secreted by CSCs. The pathway of CSCs expressing TIM-3/Galectin 9 (Gal-9) expands the number of MDSCs. Exosome S100A9 enhances STAT3/NF-κB phosphorylation and production of prostaglandin E2 (PEG-E2) to promote CSCs. Collectively, these interactions reshape the tumour microenvironment and create a habitat where Treg cells and TH17 cells support CSCs, the latter via IL-17 production

The activation of natural killer group 2 member D (NKG2D) receptor expressed on the surface of NK cells promote the lysis of MHC-I negative CSCs by a non-APC dependent mechanism [133]. NK cells expressing NKG2D can mediate the lysis of MHC-I negative colonic CD133^+^CD44^+^ CSCs [134], and NK cells expressing NKp30 and NKp44 can directly target and eradicate MHC-I negative CD24^+^ CSCs in ovarian cancer [135]. CSCs upregulate HLA-G expression, which interacts with the NK cell inhibitory ligands killer cell immunoglobulin-like receptor 2DL4 (KIR2DL4) and natural killer group 2 member A (NKG2A), making them become less sensitive to NK cell-mediated lysis by inhibiting NK cell activation [136–138]. In addition, CSCs expressing SOX2/SOX9 downregulate NKG2DL expression and protect them from NK cell-mediated immune clearance [139]. CSCs develop therapeutic resistance to NK cell-based immunotherapy by upregulating MHC-I molecules, which eventually leads to tumor recurrence [140]. Therefore, understanding the potential mechanism driving NK cell-mediated recognition and elimination of CSCs can probably provide opportunities for anti-CSC targeted immunotherapies (Fig. 1).

### Targeting CSC-immune cells therapy

From our perspective, CSCs and the tumor immune system an inextricable linked. CSCs can create their TME after cross-talk with immune cells, thus, promoting tumor immunosuppression and immune escape. This research demonstrates the therapeutic potential of focusing on CSC-TAM, CSC-T cell and CSC-MDSC cross-talk [141]. TAM can increase the increased expression of hyaluronic acid (HA) from CSCs in human neck squamous carcinoma (HNSCC); thus, targeting TAMs to inhibit CSC function is a viable option [142]. CSCs suppress T cell function by secreting cytokines (TGF-β1, CCL2 and Tenascin-C (TNC)) and exosomes to promote bone marrow-derived macrophage (BMDM) activation. MDSC promotes CSC stemness and inhibits T cell activation in breast cancer through the STAT3 signaling pathway [143]. MDSC also increases CSC stemness and PD-L1 expression in epithelial ovarian tumor cells by producing PGE2 [144]. MDSC also promotes the stemness of CSCs in ovarian cancer through triggering the CSF2/p-STAT3 signaling pathway. Therefore, it is considered that targeting MDSC and the CSF2/p-STAT3 signaling pathway can improve the efficacy of conventional therapies [145]. These preclinical studies show that targeting CSC-immune cell cross-talk has therapeutic potential in the treatment of cancer patients.

### Targeting CSC-CAR-T cells therapy

TILs are isolated from a patient, cultured with IL-2, tested for their ability to recognize tumor-specific antigens, and then reinfused into the same patient [146]. T cells have been reprogrammed into chimeric antigen receptor (CAR) T cells through the use of artificially designed CARs and gene editing techniques, allowing T cells to more effectively lyse tumor cells. CAR-T cells first showed promise in hematological tumors, then in a variety of other solid tumors [147]. CAR-T cells currently lack unique and specific targets. Several issues concerning the effective concentration and persistence of CAR-T cells in the target region remain unresolved [148, 149]. Current CSC CAR-T cell therapy experimental studies primarily involve in vitro coculture systems and preclinical studies; more clinical studies are needed in the future to demonstrate its efficacy alone or in combination with other tumor-targeted therapies.

CAR-T therapies have a distinct structure, the single-stranded variable fragment (scFv), which recognizes cell surface antigens directly and specifically without relying on MHC down-regulation [150]. The identification of CSC surface markers such as EpCAM, CD44, and CD133 has caused the identification of specific therapeutic targets for inhibiting tumor recurrence and metastasis [151]. Furthermore, CSC expressed molecular markers like epidermal growth factor receptor variant III (EGFRvIII), human epidermal growth factor receptor 2 (HER2) as well as chondroitin proteoglycan sulfate 4 (CSPG4) provide therapeutic targets for inhibiting tumor recurrence and metastasis [152–154]. CAR-T cell development targeting CSC molecular markers has so far demonstrated therapeutic efficacy. As shown in Table 2, CD133, EpCAM and ALDH have been adopted for CSC-directed immunotherapy, and the majority of them are recruited. Because the presence of CSCs in TME prevents autologous cells and T cells receiving CAR-T therapy from directly destroying tumor cells, a combination of CSCs-targeted CAR-T therapy and CSCs-targeted TME strategies may improve prognosis. Current research indicates that increased PD-L1 expression in CSCs promotes the occurrence and progression of TME [155]. The binding of PD-L1 to PD-1 on activated T cells can inhibit CAR-T cell function, resulting in CAR-T cell failure [156]. Therefore, CSCs targeted therapies combined with FDA-approved PD-1/PD-L1 checkpoint inhibitors [157] or dual CTLA-4 blockade provided significant anti-tumor effects and CSC eradication [157, 158]. Therefore, combining a-PD-L1 and a-CTLA-4 inhibitors with CAR-T cells that target CSCs may become an efficient immunotherapeutic strategy for treating cancer patients.

**Table 2:** CSCs targeted CAR-T cells therapy in clinical trials

Trial	Solid Tumor	Phase	Enrollment	NCT Number	Current status
CD133 CAR-T	Advanced malignancies	I/II	20	NCT02541370	Completed
MUC1 CAR-T/PD-1 KO	Advanced esophageal cancer	I/II	20	NCT03706326	Recruiting
EGFR IL-12 CAR-T	Metastatic colorectal cancer	I	20	NCT03542799	Not yet recruiting
MESO CAR-T	Refractory–relapsed ovarian cancer	I/II	20	NCT03916679	Recruiting
MESO-19 CAR-T	Metastatic pancreatic cancer	I	4	NCT02465983	Completed
MOv19-BBz CAR -T	Recurrent high-grade serous ovarian cancer	I	18	NCT03585764	Recruiting
LeY CAR-T	Advanced cancer	I	30	NCT03851146	Recruiting
EpCAM CAR-T	Recurrent breast cancer	I	30	NCT02915445	Recruiting

### Targeting CSC-NK cells therapy

The targeting of NK cells to CSCs highlights the translational potential of NK immunotherapy as a treatment for solid malignancies [159, 160]. Moreover, CD34^+^ AML stem cells suppress NKG2DL expression via poly-ADP-ribose polymerase 1 (PARP1), implying that NKG2DL mediates immune evasion of NK cell depletion and that genetic or pharmacological inhibition of PARP1 inhibits NKG2DL expression in CD34^+^ AML stem cells. This causes NKG2DL re-expression on the surface of AML stem cells, making them re-sensitive to NK cells [161]. Melanoma CCR7^+^ CSCs have increased NKp30/NKp46 ligand expression while decreasing MHC-I expression, making them become vulnerable to NK cell-mediated cytotoxicity [162].

The combination of autologous NK cell enhancement and engineered CAR-NK cells can target CSCs with increased affinity. Activation of NK cells by cytokines induction into killer (CIK) cells can resensitize NK-resistant CSCs, but the cytokine dose must be adjusted to avoid the expansion of immunosuppressive Tregs [163]. CIKs with anti-tumor activity recognize NKG2D and kill CSCs [164], combining CIK-mediated tumor cell killing with artificially engineered CAR cells. CAR-CIKs can be created to target CSC antigens including CD44v6 and CSPG4 [165, 166]. These CAR-CIKs are effective at eliminating CSCs both in vitro and in vivo, but more clinical trials are required to assess the synergistic effect with other therapeutic strategies. The therapeutic effect of breast CSCs has been significantly improved by using CAR-NK cells to eliminate the EGFR in the mouse in vivo model [167]. The same cytokine IL-15 can induce CAR-NK cell expansion in vivo and has a high affinity for EpCAM^+^ CSC [168]. Understanding the underlying mechanisms of NK cell-mediated CSC recognition and clearance may thus lay the groundwork for a new generation of CSC-targeted immunotherapy.

### Targeting CSC-DC vaccines therapy

Tumor vaccines can stimulate the human immune system, inhibiting tumor growth or eliminating tumor cells. Patients can be immunized by delivering tumor antigens through various established methods. DC-based vaccines are effective against CSCs in a variety of cancers. ALDH^+^ CSC-DC vaccines can directly target ALDH upregulated by CSCs, indicating the potential for adjuvant therapy in cancer patients [158, 169]. MUC1, a transmembrane glycoprotein, is involved in CSC stemness maintenance, and CSC vaccines targeting MUC1 have been developed, primarily by activating humoral immunity to inhibit CD133^+^ CSCs [170]. At the moment, a strategy of combining CSC-DC vaccine with chemotherapeutic drugs has been proposed to make targeting CSCs more effective and safe [171], and more clinical trials are needed to prove this.

### CSCs targeted by Oncolytic Viro Therapy (OVT)

OVT has an anti-CSC effect by inducing tumor cell death and activating T cells. OVT has been shown to mediate IFN-γ release, angiogenesis inhibition and a decrease in the number of regulatory T cells in the tumor [172]. Oncolytic viruses that target specific CSC markers and signaling pathways can potentially be used as CSC therapeutics. Herpes simplex virus (HSV), adenovirus (Ads), measles virus (MV), retrovirus and vaccinia virus (VACV) have all been used in clinical trials to target CSCs. HSV has received much attention for its ability to kill tumor cells [173], with oncolytic HSV (oHSV), G207, being used in clinical advanced glioma trials, and CD133^+^ CSCs glioma cells being susceptible to tumor lysis by HSV [174]. oHSV modified by interleukin IL-12, on the other hand, will switch from a pro-tumor T helper (Th) -2 response to an anti-tumor Th-1 response [175]. Ads have the ability to infect both dividing and non-dividing tumor cells [176]. To evaluate the killing effect of conditional replication Ad (CRAd) on breast cancer, CD44^+^CD24^−^ CSCs extracted from the pleural effusion of patients with metastatic breast cancer and injected into the fat pad of SCID mice decreased after tumor formation, which may contribute to the differentiation and proliferation of CSCs to form solid tumors. Five weeks after intratumoral injection, CRAd treatment demonstrated significant anti-tumor effects [177]. MVS form syncytial bodies in neighboring cells via viral protein binding and receptor protein fusion, so oncolytic MVS (oMVS) are used to induce syncytial formation in CSCs to ensure complete tumor eradication [178, 179]. After chemotherapy, the proportion of CD44^+^CD24^–^ CSCs increased significantly, whereas oMV infection caused apoptosis of CD44^+^CD24^–^ CSCs [180]. The oncolytic potential of VACV is realized via susceptibility and oncolytic action. In the breast cancer model, mice were injected into the left and right fat pads with tumor implants containing CD44^+^CD24^–^ and CD44^+^CD24^+^ CSCs, respectively. After post-orbital delivery of VACV, the left and right breast tumors were generally suppressed, indicating that VACV could be used for systemic treatment of breast cancer [181]. At the moment, oncolytic viruses combined with standard chemotherapy have been shown to be feasible and effective in the treatment of CSCs [182]. Furthermore, the sensitivity and susceptibility of oncolytic viruses to host tumor cells remains a critical issue for oncolytic virus engineering.

## Immune checkpoint-targeted immunotherapy for CSCs

### Cancer stem cells and innate immune checkpoint

A leading conundrum is how it is probable that even a subset of patients can yield a spontaneous CD8^+^ T cell response against tumor-associated antigens, obviously in the lack of pathogen involvement. Moreover, this can narrow to a question of mechanisms of sterile immunity and indicate the likely participation of stress-associated or damage-associated molecular patterns triggering innate immune activation [183]. CD47 is a transmembrane protein belonging to the immunoglobulin superfamily [106, 184–187]. The binding of CD47 to SIRPα generates a "do not eat me" signal [188–193]. Increased CD47 expression in tumors to evade immune surveillance by macrophages has also been associated with poor clinical prognosis [194]. Blockade of CD47-SIRPα interaction in cancer induces the activity of the innate immune system and increases phagocytosis of CSCs by macrophages [195]. By extending the potential clinical application of CD47 blockade combined with CAR-T cells to a wider range of malignancies [195], these treatment modalities can reduce the survival of CSCs and thereby prevent tumor recurrence. Therefore, targeting CD47 have emerged as an effective therapeutic strategy for cancer.

### Cancer stem cells and adaptive immune checkpoint

CSCs avoid immune attacks by reducing the expression of adaptive immune checkpoints, which can directly contribute to immune activation. PD-1-PD-L1 axis refers to one of the immune checkpoints that can enable tumor cells to evade immune attack from PD-1^+^ T cells [196]. Following interaction with PD-L1 and PD-L2, PD-1 inhibits T cells mediated immune responses and subsequently induces IL-10 production by the tumor [197–202]. PD-L1 expression has also been detected in CSCs [203–207]. Activation of PI3K/AKT and mTOR signaling pathways by PD-L1 is a key cellular process that maintains the pluripotency of CSCs and detects the differentiation fate of CSCs [208]. Activation of the EMT/STAT3 signaling axis induces PD-L1 expression on CSCs, enabling them to circumvent immune attacks [209]. Therefore, specific targeting of the PD-1-PD-L1 axis with monoclonal antibodies may serve as a potential therapeutic intervention for CSCs [127, 210].

Cytotoxic T lymphocyte-associated protein 4 (CTLA-4) is a member of the immunoglobulin superfamily and encodes a protein that inhibits the overactivation of T cells [211, 212]. Upregulation of CTLA-4 on Tregs plays an immunomodulatory role in suppressing overreactive T cells and protecting tissues from immune-mediated damage [213, 214]. CD28 on T cells interacts with CD80 and CD86 on the surface of APC and can offer a costimulatory signal for T cell activation [214]. Monoclonal antibody targeting CTLA-4 can trigger an anti-tumor immune response [215]. Combination therapy with specific antibodies to CTLA-4 and PD-1 may be an effective way to treat patients with tumors [216].

T cell immunoglobulin mucin receptor 3 (TIM-3) regulates immune responses mediated by various kinds of immune cells, like CD8^+^ T cells, Foxp3^+^ Treg cells and macrophages [217–222]. TIM-3 plays the role of an immune checkpoint on T cells driving immune tolerance, and thus the defective expression of this checkpoint contributes to the development of autoimmune diseases and tumors [223–225]. TIM-3 is overexpressed on AML LSCs [226, 227] and CSCs in many solid tumors [112, 228–233]. TIM-3^+^Foxp3^+^ Treg cells express IL-10 and upregulate CTLA-4 and PD-1 expression, and these cells display more tumor suppressive function than TIM-3^–^Foxp3^+^ Treg cells [234, 235]. IL-12 and IL-18 mediate the expression of TIM-3 on NK cells and inhibit the anti-tumor activity of NK cells [236–238]. There is evidence that TIM-3 expressed on T cells interacts with Gal-9 on CD11b^+^Ly6G^+^ MDSCs to induce the proliferation of MDSCs, creating an immunosuppressive environment to regulate immune responses [239]. Treatment of Th-1 cells by TIM-3 monoclonal antibodies induces immune responses against tumor cells by modulating the ERK signaling pathway in Th-1 cells [240].

**Fig. 2:**
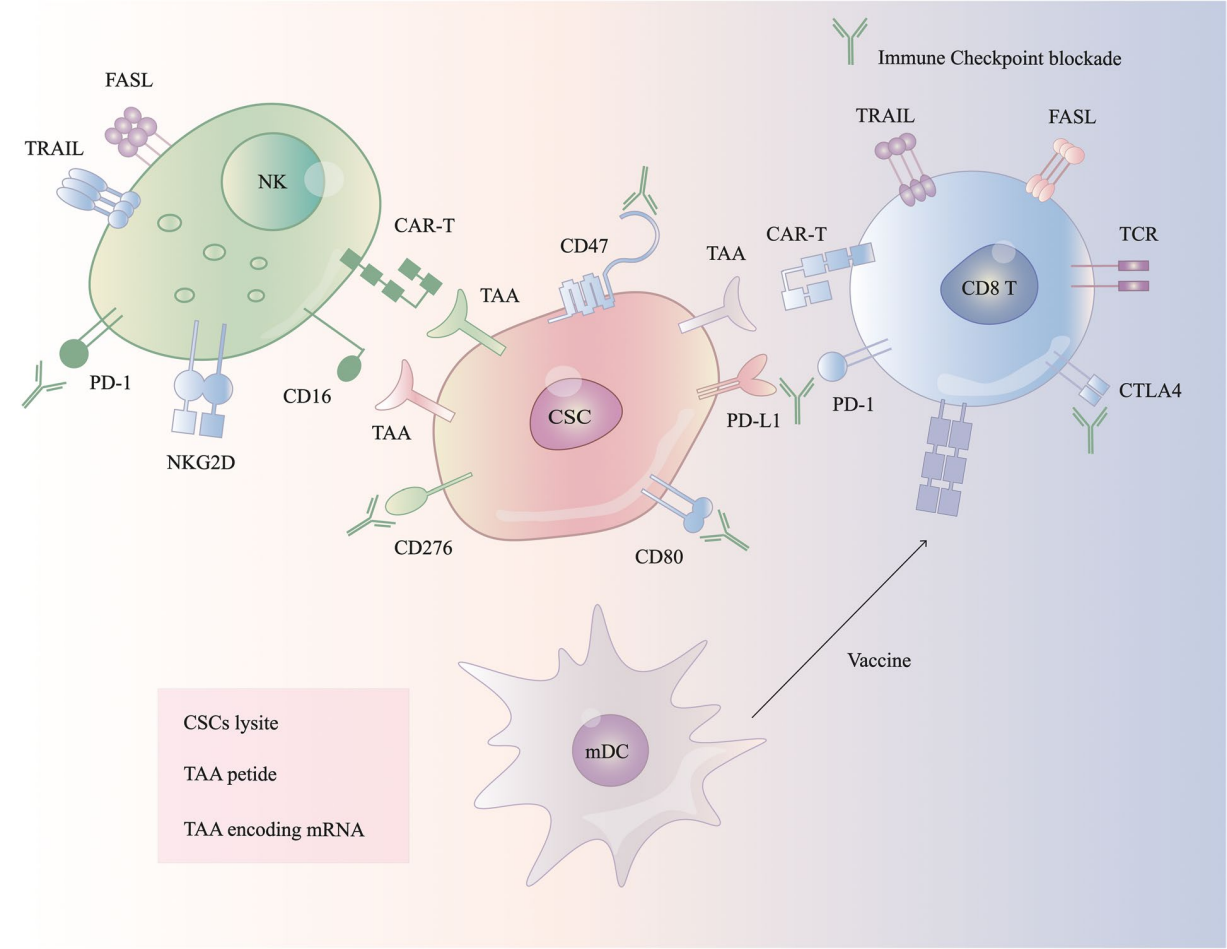
Immune checkpoint targeting CSCs. Administrated NK cells or CAR NK cells target TAAs on CSCs. Ex vivo maturation of DCs exposed to CSCs-lysate/TAAs/peptides produce a vaccine that after administration arm the cytotoxic T cells in an MHC-1-TCR-dependent manner for targeting specifc CSCs. Antibodies targeting immune checkpoint molecules such as PD1/PDL1, CD276, and CTLA4 could improve the anticancer immune responses. Anti-CD47 antibody sensitizes CSCs to cell-mediated phagocytosis. FASL, FAS ligand; mDC, mature DC; TRAIL, TNF-related apoptosis-inducing ligand

Lymphocyte-activation protein 3 (LAG-3), an immunoglobin (Ig) superfamily protein, is denoted on NK cells, activated CD4^+^ and CD8^+^ T cells and Treg cells [241]. Galectin-3, a carbohydrate-binding protein, is highly expressed in breast, gastric, colorectal and ovarian cancers [242]. Interaction of LAG-3 with MHC class II molecules can inhibit the function of melanoma-infiltrating lymphocytes and enables tumors to escape recognition and lysis by immune cells [243, 244]. Interaction of Galectin-3 on tumor cells with LAG-3 on CD8^+^ T cells inhibits anti-tumor immune responses [245]. In glioblastoma multiforme (GBM), expression of galectin-3 on CSCs mediates immunosuppression by inducing T cell apoptosis [246]. CSCs mediated activation of LAG-3 and PD-1/PD-L1 signaling pathways synergistically hinders IFN-γ and TNF secretion from CD8^+^ T cells; therefore, combined blockade of LAG-3 and PD-1 is likely to activate T cells more potently in clinical settings [247, 248] (Fig. 2).

### Targeting CSCs on the efficacy of immune checkpoint inhibitor therapy

Evaluating CSCs regulation with immune checkpoints and their relationship to tumor recurrence is an issue that needs to be addressed further. Alternative checkpoints, such as v-domain immunoglobulin inhibitory T-cell activation (VISTA) and indoleamine 2, 3-dioxygenase 1 (IDO1), inhibit the tumor-killing function of T cells in addition to PD-L1 and CTLA-4 [249]. The intrinsic mechanism of tumor resistance caused by alternative checkpoints and PD-1 treatment must be clarified [250]. Anti-PD-L1 antibodies have limited specificity, and PD-L1 heterogeneity is caused by differences in affinity or target epitopes [251]. On the other hand, patients with androgen receptor prostate cancer did not express PD-1, PD-L1 or CTLA-4, whereas the B7-H3 is highly expressed [252] and inhibits cytotoxic T cell activity [253]. Immune checkpoint heterogeneity will influence immune checkpoint inhibitor (ICI) therapy response and can be applied to be a tool to identify appropriate targeted checkpoints in different tumor types. In addition, based on a better understanding of CSC surface biomarkers, obvious progress has been made in the development of antibodies that target CSCs (Table 3).

**Table 3:** Targeting agents on the efficacy of CSCs associated surface markers in clinical trials

Antibody target	Drug name	Solid Tumor	Phase	Enrollment	NCT number	Current status
CD47	TTI-621	Solid tumor	I	260	NCT02663518	Recruiting
	Hu5F9-G4	Solid tumor	I	88	NCT02216409	Completed
	IBI188	Advanced malignancies	I	42	NCT03763149	Recruiting
	AO-176	Solid tumor	I	90	NCT03834948	Recruiting
	SRF231	Solid tumor	I	148	NCT03512340	Recruiting
	Bivatuzumab mertansine	Metastatic breast cancer	I	24	NCT02254005	Completed
CD44	RO5429083	Malignant solid tumors	I	65	NCT01358903	Completed
	SPL-108	Ovarian cancer	I	18	NCT03078400	Recruiting
PD-1	Nivolumab	Glioblastoma multiforme	II	29	NCT02550249	Completed
	Pembrolizumab	Glioblastoma multiforme	II	80	NCT02337491	Completed
	Durvalumab	Solid tumors	II	124	NCT02403271	Completed
PD-L1	Atezolizumab	Non-small-cell lung cancer	III	1225	NCT02008227	Completed
	Avelumab	Recurrent glioblastoma	II	52	NCT03291314	Completed
TIM3	Sym023	Solid tumors	I	48	NCT03489343	Recruiting
CD70	Varlilumab (CDX-1127)	Solid tumors	II	175	NCT02335918	Completed
LAG3	Sym022	Solid tumors	I	30	NCT03489369	Recruiting
CD70/LAG3	MGD013	Solid tumors	I	255	NCT03219268	Recruiting
EpCAM/CD3	Catumaxomabr (emovab)	Ovarian cancer	II	44	NCT00189345	Completed
CD44V6	AMC303	Solid tumor	I	55	NCT03009214	Recruiting
CTLA-4	Ipilimumab	Non-small-cell lung cancer	II	24	NCT01820754	Completed

In terms of anti-tumor and immunotherapy efficacy, CSCs represent a novel target for cancer treatment. Because CSCs can continue to develop into drug-resistant tumors even after conventional treatment. CSC therapy can therefore be combined with immune checkpoint inhibitor (ICI) therapy to produce a more potent antitumor effect. B-lymphoma Mo-MLV insertion region 1 (BMI1) is a critical component of polycomb reactive complex 1, which coordinates immune escape in CSCs [254]. The proportion of BMI1^+^ CSCs in HNSCC increased significantly after anti-PD-1 and cisplatin combination therapy, whereas BMI1 inhibition resulted in the elimination of these CSCs and a significant increase in CD8^+^ T cell infiltration. Depletion of BMI1^+^ CSCs may thereby be an efficient strategy for improving anti-PD-1 therapy efficacy and preventing tumor recurrence [255]. Depletion of BMI1^+^ CSCs may thus be an effective strategy for improving anti-PD-1 therapy efficacy and preventing tumor recurrence [255]. Metformin directly kills cancer stem cells [256] while improving anti-PD-1 therapy efficacy [257]. In addition, the two functions are linked.

TME complex components contribute to CSC dedifferentiation, causing them to intervene in tumor immunogenicity rather than tumor immunosuppression [258]. There exists a significant positive relationship between tumor immunogenicity and ICI therapy efficacy, but more research is needed in this area, particularly in different types of cancer. The concept of tumor heterogeneity leading to a low immune response to tumors is important in clinical evaluation [259]. Intertumor or intratumor heterogeneity is thought to be an impediment to tumor targeted therapy [260]. Tumor heterogeneity and cancer stem cell plasticity are linked, and it is thought to be an emerging marker related to cancer invasion [261]. Tumor heterogeneity is caused by the state of the complex tumor immune microenvironment [262], tumor mutation reflects immune characteristics [263] and represents tumor sensitivity to anti-PD-1 treatment [264].

CSCs can transition from epithelial to mesenchymal cells [265], which is due to their epithelial-mesenchymal plasticity (EMP) [266]. The phenotype of EMT is strongly associated with elevated levels of immune checkpoint expression (PD-1, PD-L1, CTLA-4 and TIM-3). Therefore, EMT characteristics have been proposed as predictors of response to ICI therapy [267]. Zinc finger E-box binding homeobox 1 (ZEB1) is a critical transcription factor in EMT that connects CSCs to EMT [268]. ZEB1 was also linked to increased PD-L1 expression and tumor killing by T cells [269]. PD-L1 on the surface of CSCs is downregulated after they transform to the MET phenotype, resulting in increased sensitivity to TIM-3 targeted therapy.

CSCs make a critical function in the promotion of angiogenesis in solid tumors. Anti-angiogenic inhibitors, like cabozantinib and regorafenib, are currently approved for the treatment of HCC after sorafenib failure [270, 271]. Ramucirumab, an anti-VEGF antibody, has also been approved for patients undergoing unresectable HCC who have failed sorafenib treatment [272, 273]. These anti-vascular therapies, when combined, may have anti-tumor effects by targeting CSCs. Furthermore, the relationship between CSCs and angiogenesis promotes tumor cell immune escape. Therefore, immunotherapy combined with a VEGF antagonist is a novel approach with clinical potential [274]. A phase III trial (IMbrave150) recently found that combining atezolizumab (a PD-L1 inhibitor) and bevacizumab (an anti-VEGF antibody) led to improved overall survival and progression-free survival in patients undergoing unresectable HCC (NCT03434379) [275]. Therefore, the FDA approved atezolizumab and bevacizumab as the most recent first-line systemic treatment for patients with unresectable HCC [275]. Furthermore, the REGONIVO trial (NCT03406871) showed that combining nivolumab (a PD-L1 inhibitor) and regorafenib (an anti-VEGFR antibody) resulted in responses in patients suffering from gastric and colorectal cancer [276]. Finally, the combination of immune checkpoint inhibitors and anti-angiogenic inhibitors may result in CSC depletion.

## Secretome-targeted immunotherapy for CSCs

### CSCs and their EVs are essential for the progression of cancer

Cell-to-cell communication occurs through different pathways, such as tunneling, microtubules reorganization and direct intercellular connections created by connexin channels; while extracellular vesicles (EVs) are increasingly recognized as an important mediator of intercellular communication [277]. EVs mediate intercellular transport of biomolecular cargo, such as non-coding nucleic acids, mRNA, proteins, metabolites and intact organelles [278]. EVs can influence the proliferation and energy metabolism of cancer cells as well as the components of the tumor microenvironment [279, 280]. EVs also result in the dedifferentiation of cancer cells into the CSC state.

Given the considerable heterogeneity of CSCs and EVs in various cancers, the impact of these cells and EVs secreted by these cells is also widespread, yet CSCs share some properties with cancer cells that help develop resistance to immunotherapy by evading immune surveillance [281]. Numerous cellular processes contribute to the maintenance of the specific functions of CSCs, including autophagy and EVs secretion [282], with autophagy contributing to the transport of cellular proteins as well as the secretion of EVs [282, 283]. Targeting EV secretion could become a possible therapeutic strategy for anti-tumor therapy [282, 284]. EV-mediated communication between non-CSCs and CSCs are essential for adaptation to the ecological niches [285, 286]. CSCs-derived EVs are engaged in tumor metastasis, resistance to therapy, angiogenesis, maintenance of stemness and immunosuppression [287, 288]. The fusion of CSC-derived EVs with macrophages and other immune cells mediates immunosuppression through the release of proteins and miRNAs [289–294].

### The EVs: the role of CSCs and immune cells

**Fig. 3:**
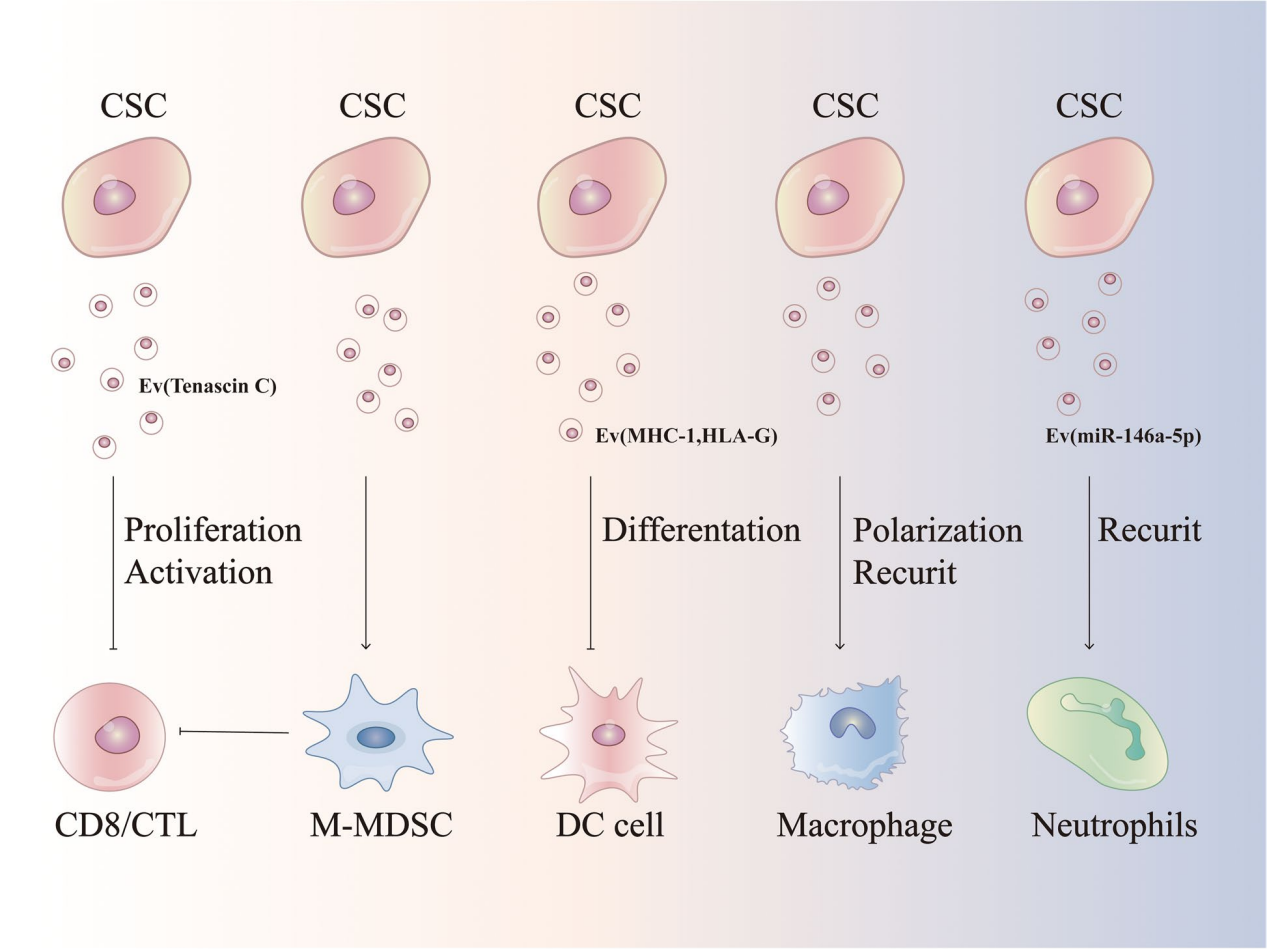
Crosstalk between CSCs and TME cells. CSCs secrete exosome regulated infltrating immune cells (IICs), MDSC, DC, macrophage and neutrophils to the TME. Cell–cell interactions in TME contribute to the development of cancer. The mechanism of interaction between CSCs and tumor-infiltrating immune cells is not only through immune targets, but can likewise be through exosomes that enable a large exchange

Cell-to-cell interaction in the TME contributes to carcinogenesis [295]. The interaction between CSCs and immune cells is mediated not only through immune targets, but also through EVs that enable the transfer of large biomolecular cargos among different types of cells [295, 296]. Exosomes, with an average diameter of -100 nm, are a subset of EVs. Interaction of exosomal tenascin C with integrins α5β1 and αvβ6 on T cells attenuates p-mTOR signaling [297, 298]. CSCs-derived exosomes can also suppress T cell function by inducing bone marrow-derived myeloid cells [299]. EVs released from CD105^+^ CSCs inhibit dendritic cell maturation and T cell-mediated immune responses [99]. Furthermore, the transfer of CSCs-derived exosomes into monocytes triggers monocyte agonist protein reorganization, induces monocyte differentiation into immunosuppressive M2 macrophages, and increases PD-L1 expression on CSCs via the STAT3 signaling pathway [300]. Glioblastoma is infiltrated with numerous microglia, and cross-talk between glioblastoma and microglia induces immunosuppressive TME in tumor mass [301]. Following coculture with microglia cells, glioma CSCs release exosomes carrying lncRNA MALAT1, which induces secretion of IL-6 and TNF-α from LPS-stimulated microglia cells. Colorectal CSCs derived exosomes activate NF-κB signaling cascade in neutrophils inducing IL-1β expression [302, 303] (Fig. 3).

### EVs-based therapeutic strategies for targeting CSCs

Tumor therapy mediated by exosomes targeting CSCs has been revealed to be extremely efficient in the clinical trials. More precise targeted therapy can be achieved by improving existing exosome engineering technology to target the unique markers of CSCs. CD44 is highly expressed in metastatic HCC CSCs, and anti-CD44 antibody-coated liposomes can directly deliver doxorubicin to CSCs [304]. The anti-CD44 antibody can cause apoptosis in CD90^+^ HCC CSCs. Similarly, anti-CD44 antibody-coated exosomes can cause CSCs to die [305]. In addition, anti-CD44 antibody-coated exosomes can also be used for drug delivery. As a result, other CSC markers including EpCAM, CD133 and CD24 can be applied as targeting candidates to enhance the efficacy of engineered exosomes targeting CSCs. Because CSCs surface markers may be denoted on normal cells, antibody-coated exosomes must be engineered to enhance their targeting efficiency to cancer stem cells and thus reduce side effects on normal cells.

Compared to synthetic nanoparticles, nanotechnology-based drug delivery systems are more biocompatible, biodegradable, less toxic and immunogenic [306–309]. Thus, exosome-based nanocarrier drug delivery technologies with advanced targeting capabilities have been developed, and they show great promise in targeting CSCs [310–312]. The development of exosome-nanoparticle technology based on EVs as a drug delivery vehicle targeting CSCs will aid in improving the anti-tumor immune response [313, 314]. A recent study found that biocompatible tumor cell-exocytosed exosomes encapsulated doxorubicin-loaded mimetic porous silica nanoparticles (PSiNPs) have the potential to be enriched inside CSCs, resulting in CSC eradication [315]. Finally, exosome engineering approaches are likely to improve the efficacy of CSCs targeted therapies.

## Conclusions

Cancer immunotherapy is adopted to either suppress tumor growth or remove tumor cells through activating the immune system; consequently, cancer immunotherapy shows great potential in treating malignant diseases from different cancer types. CSCs can suppress the immune response by recruiting immunosuppressive cells (TAM and Tregs); thus, promoting the establishment of an immunosuppressive TME. CSCs can also impair NK cell function by expressing specific ligands. In this outlook, we demonstrate the mechanism by which CSCs communicate with immune cells in the tumor microenvironment in a variety of cancer types. Therefore, there is a need to find new strategies to target CSCs through immunotherapeutic approaches.

CSCs evade immune surveillance through various immune checkpoints, which are expressed at higher levels in CSCs. CSCs express CD47, CTLA4, PD-L1, TIM-3 and LAG3, which promote immune evasion in the malignant environment and maintain tumor survival. In addition, CSCs orchestrate the tumor microenvironment by releasing immunosuppressive cytokines and growth factors. CSCs can also modulate the immune microenvironment of tumors through the excretion of EVs; thus, further understanding of the molecular mechanism driving anti-tumor immune response is a prerequisite to develop new anti-tumor therapies with higher efficacy.

To achieve desired therapeutic goals, CSC protocols need to be optimized in immunocompetent preclinical models, and the contact of CSCs with the immune system is needed to be studied using models that rigorously validate functional and phenotypic characteristics of CSCs. Traditional two-dimensional coculture experiments have been performed to clarify the mechanisms promoting CSC characteristics in the TME; unfortunately, two-dimensional models do not allow for the observation of dynamic cellular interactions in real time. Three-dimensional coculture systems enable us to better visualize the complex interactions between CSCs and immune effectors. The current emerging 3D cell coculture models are represented by organoids that closely resemble tumor microenvironments, including ecological niches that nurture host CSCs. With the rapid development of single-cell spatial analysis, it would be possible to visualize the complex interactions involving different types of immune cells and CSCs.

## Data Availability

Not applicable.

## Abbreviations

CSCs: Cancer stem cells
TME: Tumor microenvironment
EVs: Extracellular vesicles
TIME: Tumor immune microenvironment
AML: Acute myeloid leukemia
PD-L1: Programmed death-1/programmed cell death ligand
CD47: Cluster of differentiation 47
TIM3: T cell immunoglobulin and mucin-containing domain-3
LAG3: Lymphocyte activation gene 3
CTLA4: Cytotoxic T-lymphocyte antigen-4
ILs: Interleukins
MMPs: Matrix metalloproteinases
VEGF: Vascular endothelial growth factor
TGF-β1: Transforming growth factor beta 1
Hh: Hedgehog
NF-κB: Nuclear factor kappa B
YAP: Yes-associated protein
HCC: Hepatocellular carcinoma
LIF: Leukemia inhibitory factor
H3K27 me3: Lysine 27 of histone H3
IHH: Indian hedgehog
SHH: Sonic hedgehog
TAZ: Transcriptional coactivator with PDZ-binding
ERK1/2: Extracellular signal-regulated kinase 1/2
ECM: Extracellular matrix
αVβ3: Alphavbeta3
CAF: Cancer-associated fibroblasts
HGF: Hepatocyte growth factor
DLL4: Notch ligand Delta-like ligand 4
DCs: Dendritic cells
APCs: Antigen-presenting cells
TAAs: Tumor-associated antigens
MHC: Major histocompatibility complex
HLA-G: Human leukocyte antigen G
STAT3: Signal transducer and activator of transcription 3
CXCL: C-X-C motif chemokine ligand
DCregs: Regulatory dendritic cells
CXCR: C-X-C motif chemokine receptor
TAMs: Tumor-associated macrophages
EMT: Epithelial-mesenchymal transition
MDSCs: Myeloid-derived suppressor cells
mTOR: Mammalian target of rapamycin
Gal-9: Galectin 9
LSCs: Leukemic stem cells
NF-κB: Noncanonical nuclear factor-kappaB
PEG-E2: Prostaglandin E2
COX-2: Cyclooxygenase 2
HNCCs: Human neural crest cells
NLRC5: NLR Family CARD Domain Containing 5
NKG2D: Natural killer group 2 member D
KIR2DL4: Killer cell immunoglobulin-like receptor 2DL4
NKG2A: Natural killer group 2 member A
HA: Hyaluronic acid
HNSCC: Human neck squamous carcinoma
TNC: Tenascin-C
BMDM: Bone marrow-derived macrophage
CAR: Chimeric antigen receptor
scFv: Single-stranded variable fragment
EGFRvIII: Epidermal growth factor receptor variant III
HER2: Human epidermal growth factor receptor 2
CSPG4: Chondroitin proteoglycan sulfate 4
PARP1: Poly-ADP-ribose polymerase 1
CIK: Cytokines induction into killer
OVT: Oncolytic Viro Therapy
HSV: Herpes simplex virus
Ads: Adenovirus
MV: Measles virus
VACV: Vaccinia virus
oHSV: Oncolytic HSV
Th: T helper
CRAd: Conditional replication Ad
oMVS: Oncolytic MVS
TCR: T cell receptor
Ig: Immunoglobin
GBM: Glioblastoma multiforme
VISTA: V-domain immunoglobulin inhibitory T-cell activation
IDO1: Indoleamine 2, 3-dioxygenase 1
ICI: Immune checkpoint inhibitor
BMI1: B-lymphoma Mo-MLV insertion region 1
EMP: Epithelial-mesenchymal plasticity
ZEB1: Zinc finger E-box binding homeobox 1
PSiNPs: Porous silica nanoparticles
